# Patient Satisfaction With Perioperative Services and Associated Factors in Ethiopia: Systematic Review and Meta-Analysis

**DOI:** 10.2196/84457

**Published:** 2026-08-07

**Authors:** Melesse Abiye Munie, Tegene Atamenta Kitaw, Aregash Birhane Terefe, Alemu Birara Zemariam, Molalign Aligaz Adisu, Molla Azmeraw, Tesfaye Engdaw Habtie, Betelhem Walelgn Dagnaw, Abebe Merchaw Faris, Tilahun Wodaynew, Amsalu Baylie Taye, Gebrie Kassaw Yirga, Yabibal Asfaw Desro

**Affiliations:** 1 Department of Nursing, College of Health Sciences, Woldia University, PO Box 400, Woldia, Ethiopia, +251-33-540-0686; 2 Department of Pediatrics and Child Health Nursing, College of Health Sciences, Woldia University, Woldia, Ethiopia; 3 Department of Emergency and Critical Care Nursing, College of Health Sciences, Woldia University, Woldia, Ethiopia; 4 Department of Surgical Nursing, College of Health Sciences, Woldia University, Woldia, Ethiopia

**Keywords:** patient satisfaction, perioperative care, surgical patients, Ethiopia, systematic review, meta-analysis, associated factors, prevalence

## Abstract

**Background:**

Patient satisfaction is a key indicator of health care quality, and it guides improvement efforts. Although many local studies have examined perioperative patient satisfaction in Ethiopia, there is no comprehensive national synthesis. This gap limits the development of targeted strategies to enhance patient care.

**Objective:**

The aim of this systematic review and meta-analysis is to determine the pooled prevalence of patient satisfaction with perioperative services in Ethiopia and identify associated factors.

**Methods:**

This study included all observational research articles on patient satisfaction with perioperative services in Ethiopia. A multidatabase search strategy, incorporating PubMed/MEDLINE, HINARI, Web of Science, Cochrane Library, African Journals Online, and Scopus, was used alongside a gray literature search to identify all Ethiopian studies on perioperative satisfaction available before January 1, 2024. The Newcastle-Ottawa Scale was used to assess the quality of the studies. To assess heterogeneity, subgroup analyses were conducted, and *I*² statistics were calculated. This study used funnel plots, the Egger test, and a nonparametric trim-and-fill analysis to assess publication bias. A sensitivity analysis was also used to identify any influential studies. Univariate meta-regression examined the association between study-level covariates and perioperative satisfaction.

**Results:**

This review included 21 studies comprising 6858 participants. Overall satisfaction with perioperative services was expressed by 5072 participants (73.96%, 95% CI 68.84%-79.08%; *I*²=96.56%). Factors significantly associated with higher satisfaction included effective postoperative pain management (adjusted odds ratio [AOR] 2.23, 95% CI 1.56-2.90), illiteracy (AOR 3.18, 95% CI 1.23-5.13), primary school education (AOR 6.55, 95% CI 3.61-9.49), local anesthesia use (AOR 2.80, 95% CI 2.03-3.57), and history of prior surgery or anesthesia (AOR 2.76, 95% CI 1.51-4.01).

**Conclusions:**

This study found that the pooled prevalence of patient satisfaction with perioperative services in Ethiopia was 73.96% (5072/6858 participants). Postoperative pain management, illiteracy, primary school, local anesthesia, and a history of surgery or anesthesia were significantly associated with patient satisfaction with perioperative services. Health care facilities should focus on providing effective postoperative pain management, clear information about perioperative services, and training for surgical and anesthesia teams to boost patient satisfaction with perioperative services in Ethiopia.

## Introduction

Patient satisfaction is a subjective measure that reflects how well patients’ health care experiences align with their expectations and needs. It is an essential indicator of the quality of health care services, particularly in the perioperative setting, where patients undergo significant physical and emotional changes [1]. Patient satisfaction is a vital goal in health care, acting as both an indicator of care quality and a catalyst for improvement. By assessing satisfaction, providers can pinpoint areas needing enhancement, which leads to better patient outcomes and adherence to treatment protocols [2]. High patient satisfaction is correlated with improved quality of perioperative care and a reduction in patient readmissions, mortality rates, and hospital stays [3].

Perioperative patient satisfaction is a critical outcome that enhances recovery and trust. Perioperative care integrates management across the preoperative, intraoperative, and postoperative phases. This multidisciplinary approach faces challenges, including patient anxiety, pain control, and communication issues. Addressing these challenges through holistic strategies, which commonly prioritize communication, pain management, and emotional support, can enhance perioperative patient satisfaction [4]. Effective perioperative care requires a multidisciplinary approach involving collaboration among surgeons, anesthetists, nurses, and other health care professionals. This teamwork is essential for addressing the diverse needs of patients, particularly those with greater surgical risk [5]. Engaging patients in their care, providing them with clear information, and addressing their concerns can significantly improve their satisfaction and overall experience.

Patient satisfaction with perioperative services is significantly influenced by different factors. Therefore, understanding these factors can help health care providers enhance the quality of perioperative care and improve patient experiences. The determining factors affecting patient satisfaction with perioperative services can be health care provider–related or patient-related. Health care provider–related factors include professional and interpersonal communication skills, perioperative care, patient involvement in care, staff skills and behaviors, multidisciplinary patient care approaches, staff attentiveness and support, access to care, infrastructure, and basic facilities. Patient-related factors include sociodemographic characteristics, such as age, baseline health status, overall experience, and stage of disease; perception of a relationship of trust with health care providers; and experience of intraoperative awareness, postoperative care, nausea and vomiting, postoperative complications, and feeling involved in decisions about their care [1,4,6-9].

Several studies have assessed patient satisfaction with perioperative services. These studies reported different levels of satisfaction in various areas. The overall level of satisfaction varied in studies conducted in the countries of Australia (96.8%) [7], India (73%) [10], and Eritrea (68.8%) [11] and at Sohag University Hospital (61.9%) [12]. In Ethiopia, there is a gap in understanding patient satisfaction with perioperative services at the national level. Evidence is fragmented and inconclusive, with no comprehensive national analysis available. A systematic appraisal of the literature confirms this significant knowledge gap, highlighting both the inconsistency of existing study results and the complete absence of any systematic review or meta-analysis synthesizing findings within the Ethiopian context [13-33].

The findings of this study will help policymakers, stakeholders, and other concerned bodies identify gaps and develop strategies to increase patient satisfaction with perioperative services. Moreover, it will help improve the quality of the health care delivery system by filling the gap accordingly. Understanding patient satisfaction in Ethiopia requires a national lens because the experience of care is shaped by different challenges, including scarce resources, cultural diversity, and geographic inequality. Therefore, the aim of this systematic review and meta-analysis is to determine overall patient satisfaction with perioperative services and identify associated factors in Ethiopia.

## Methods

### Study Design

In this study, a systematic review and meta-analysis were performed to determine overall patient satisfaction with perioperative services and their significant associated factors in Ethiopia. This study was conducted in line with the recommendations of the PRISMA (Preferred Reporting Items for Systematic Reviews and Meta-Analyses) guidelines [34] (Checklist 1). This systematic review and meta-analysis has been registered on PROSPERO (CRD42024614957).

### Inclusion and Exclusion Criteria

#### Inclusion Criteria

This study considered all research articles conducted in Ethiopia that met the inclusion criteria. Articles focused on patient satisfaction with perioperative services and their associated factors in Ethiopia were included. We included primary research studies that were published in the English language (researchers are encouraged or required to publish in English to gain international recognition), used observational and quantitative methods, and were available in electronic databases up to January 1, 2024. We included gray literature from preprints and institutional repositories to capture timely and locally relevant information. This approach minimized publication bias, time-lag bias, and geographic bias while maintaining methodological rigor through consistent quality appraisal and transparent reporting.

#### Exclusion Criteria

The systematic review and meta-analysis excluded studies conducted outside surgical departments, low-quality studies, those without clearly defined outcome variables or patient satisfaction information, and those lacking full-text access for data extraction.

### Search Strategy and Sources of Information

The search strategy was adapted from and developed according to the population, exposure, outcomes, study design, and setting framework for creating MeSH terms to retrieve potential studies in databases. The population included patients who underwent surgical therapy. Exposure included descriptions of perioperative services, any specific interventions aimed at enhancing patient satisfaction, and factors influencing satisfaction, such as predictors, barriers, and determinants. The outcome included patient satisfaction with perioperative services. Study design included observational quantitative studies. The setting, or context, was Ethiopia.

To identify relevant primary studies, we created the following review questions based on the previously mentioned framework: (1) What is the pooled prevalence of patient satisfaction with perioperative services in Ethiopia? (2) What factors influence patient satisfaction in Ethiopia?

### Electronic Databases

Primary studies were subsequently found by searching PubMed/MEDLINE, HINARI, Web of Science, African Journals Online, Cochrane Library, and Scopus, as well as other Ethiopian research repositories for additional literature and sources. Data retrieval took place from January 1 to 30, 2024. In addition to our search strategy terms, we searched electronic databases using the following free-text terms: “patient satisfaction,” “perioperative service,” “perioperative care,” “preoperative care,” “postoperative care,” “associated factors,” “determinant factors,” “operating room,” “surgery,” “recovery room,” “post-anesthesia care unit (PACU),” and “Ethiopia.” Articles were searched using Boolean operator strings with “AND” and “OR.” All available articles were organized after the data were gathered and saved. We removed duplicate articles and incorporated studies focused on patient satisfaction with perioperative services and its associated factors in Ethiopia (for the full search strategy document, see Multimedia Appendix 1).

### Study Selection Process

All included studies were imported into EndNote software (version 20; Clarivate PLC), and unnecessary files were removed from the analysis. Two independent reviewers (MAM and YAD) selected articles based on this study’s objectives and eligibility criteria. They identified potentially eligible studies according to the inclusion criteria by title, abstract, and full text and, finally, screened and compiled the articles. Any disagreements between the reviewers during study selection and data extraction were resolved through discussion and consensus. If consensus could not be reached, a third reviewer (TAK) was consulted for a final decision.

### Assessment of Study Quality

The 2 independent reviewers (MAM and YAD) independently reviewed each article that fulfilled the inclusion criteria and met the objectives by using a modified Newcastle-Ottawa Scale to confirm the quality of each study [35]. Any disagreements that occurred among the reviewers were resolved by consulting a senior researcher (TAK). The assessment scale evaluates each article’s sample representativeness, adequacy, measurement tools, response rates, groups in outcomes, control of confounding variables, outcome evaluation, and statistical testing, yielding a total possible score of 10. A total score of >5 out of 10 indicated a low risk of bias, and all studies included in this systematic review and meta-analysis scored >7 out of 10 (Multimedia Appendix 2).

### Data Extraction Tools

The data extraction format was designed and implemented in Microsoft Excel. The data extraction format included author names, study area, year of publication, working unit, study population, study design, sampling technique, sample size, assessment tools, and types of services. The format also included the prevalence for each primary outcome and the adjusted odds ratio for each factor (second outcome) with the corresponding 95% CI.

### Measurement of Outcomes

This meta-analysis included 2 outcome variables. The primary outcome was overall patient satisfaction with perioperative services. The outcome was measured using validated assessment tools, such as the Leiden Perioperative Care Patient Satisfaction Questionnaire, or questionnaires adapted from validated tools. Overall patient satisfaction was categorized as satisfied or unsatisfied according to the assessment tool used in each study, based on the median or mean score. The secondary outcome was factors affecting patient satisfaction with perioperative services. Significant factors were determined by analyzing adjusted odds ratios from the included studies. For the meta-analysis, only combined results from studies that controlled for conceptually similar confounders, such as age, sex, and type of surgery, were used.

### Statistical Analysis

This meta-analysis used Stata software (version 17; StataCorp) to analyze the retrieved data. Overall prevalence was estimated using SEs, which were calculated as p = r/n and SE = √ p (1 − p)/n, where p is the proportion, r is the total population size, and n is the sample size. The results are reported as the overall prevalence of patient satisfaction with perioperative services with 95% CI, and *P*<.05 was considered statistically significant. This study also used Stata software to identify factors significantly associated with perioperative patient satisfaction. The analysis used a random-effects model with the restricted maximum likelihood method to account for each study’s variability [36]. We assessed heterogeneity using the *I*² statistic and the Cochran *Q* test. *I*² measures the proportion of variation across studies due to heterogeneity, estimating the difference observed and assigning a value from 0% to 100%. *I*^2^ values below 25% imply low heterogeneity, values between 25% and 50% indicate moderate heterogeneity, values between 51% and 74% indicate substantial heterogeneity, and values greater than 75% suggest high heterogeneity. The Cochran *Q* test was used to estimate whether the observed differences in effect size were statistically significant, which would suggest the presence of heterogeneity [37].

To determine the heterogeneity level between studies, a subgroup analysis was conducted. Publication bias was determined by checking funnel plots for asymmetry and using the Egger test and a nonparametric trim-and-fill analysis. Each of these methods can be used to identify publication bias, but the Egger test is the most objective and effective in providing precise values [38]. A sensitivity analysis was used to evaluate the robustness of each study’s results. To identify sources of heterogeneity, study-level covariates were analyzed for their effect on perioperative satisfaction via univariate meta-regression.

### Ethical Considerations

This study did not require ethics approval. The research constitutes a systematic review of existing, publicly available scientific literature. All data analyzed were extracted from previously published studies that had already obtained their own necessary ethics approvals and participant consent. Therefore, as no new human or animal subjects were involved, ethics approval for this analysis was not compulsory.

## Results

### Literature Search

**Figure 1.**
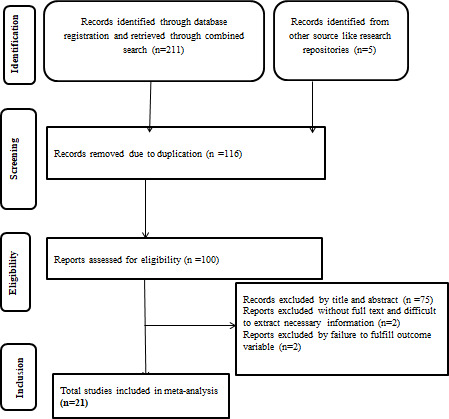
Flowchart of selection for systematic review and meta-analysis to assess patient satisfaction with perioperative services and associated factors in Ethiopia.

To conduct this systematic review and meta-analysis, we performed a search as outlined above, and 216 published records were retrieved. From the retrieved studies, a total of 116 published articles were excluded due to duplication or ineligibility by the inclusion criteria. Following a meticulous review of the titles and abstracts of the 100 remaining records, 79 articles were excluded. Finally, a meta-analysis was conducted on the remaining 21 studies (Figure 1).

### Characteristics of the Included Studies

All cross-sectional studies included in this systematic review and meta-analysis assessed patient satisfaction with perioperative services. The included studies examined 5 areas of Ethiopia: Addis Ababa [15,19,20,24,25], Amhara Region [13,14,16,17,21,27,28,30-33], Oromia Region [23,29], Tigray Region [22], and the Southern Nations, Nationalities, and Peoples (SNNP) Region [18,26]. The minimum and maximum sample sizes of the included studies were 120 and 468, respectively (Table 1).

**Table 1.** Characteristics of the 21 studies included in the meta-analysis and systematic review of patient satisfaction with perioperative services and associated factors in Ethiopia [13-33].

Author name and publication year	Area of study	Sampling technique	Assessment tools	Service provided	Sample size, n	Prevalence of patient satisfaction, n (%)	NOS^a^ score of quality assessment out of 10
Fetene et al [13] (2022)	Amhara Region	Random	Adopted validated tools	All perioperative	411	263 (63.99)	8
Endale Simegn et al [14] (2021)	Amhara Region	Nonrandom/consecutive	Adopted validated tools	Perioperative anesthetic service	398	295 (74.12)	8
Shamil [15] (2021)	Addis Ababa	Random	Adopted validated tools	Nursing care	299	196 (65.55)	9
Teshome et al [16] (2022)	Amhara Region	Nonrandom/consecutive	Adopted validated tools	Perioperative anesthetic service	387	242 (62.53)	9
Alemu et al [17] (2023)	Amhara Region	Random	Adopted validated tools	All perioperative	422	290 (68.72)	8
Ataroe et al [18] (2024)	SNNPE^b^ Region	Nonrandom/consecutive	Adapted questionnaire	Nursing care	468	372 (79.49)	9
Deressa et al [19] (2022)	Addis Ababa	Random	Adapted questionnaire	Nursing care	414	346 (83.57)	9
Kibru et al [20] (2023)	Addis Ababa	Random	Adapted questionnaire	All perioperative	287	276 (96.17)	8
Derso et al [21] (2024)	Amhara Region	Random	Adapted questionnaire	Nursing care	383	238 (62.14)	7
Benwu and Gebremedhin [22] (2019)	Tigray Region	Nonrandom/consecutive	Adapted questionnaire	Perioperative anesthetic service	120	106 (88.33)	9
Alemu [23] (2015)	Addis Ababa	Nonrandom/consecutive	Adapted questionnaire	Preoperative care	224	162 (72.32)	9
Obsa et al [24] (2017)	Oromia Region	Nonrandom/consecutive	Adapted questionnaire	Perioperative anesthetic service	184	144 (78.26)	8
Abdissa et al [25] (2023)	Addis Ababa	Random	Adapted questionnaire	All perioperative	346	286 (82.66)	9
Siraneh et al [26] (2020)	SNNPE Region	Nonrandom/consecutive	Adapted questionnaire	Perioperative anesthetic service	200	120 (60.00)	10
Ayele et al [27] (2022)	Amhara Region	Random	Adapted questionnaire	Preoperative care	404	284 (70.30)	7
Belay Bizuneh et al [28] (2020)	Amhara Region	Random	Adapted questionnaire	Postoperative pain management	418	302 (72.25)	7
Biyazin et al [29] (2022)	Oromia Region	Random	Adapted questionnaire	Preoperative care	372	160 (43.01)	8
Admass et al [30] (2024)	Amhara Region	Random	Adapted questionnaire	Postoperative pain management	424	340 (80.19)	9
Ashebir et al [31] (2024)	Amhara Region	Nonrandom/consecutive	Adapted questionnaires	All perioperative	195	168 (86.15)	8
Bayable et al [32] (2020)	Amhara Region	Nonrandom/consecutive	Adapted questionnaire	All perioperative	382	315 (82.46)	7
Demilew et al [33] (2021)	Amhara Region	Nonrandom/consecutive	Adapted Questionnaire	Perioperative anesthetic service	120	96 (80.00)	8

a NOS: Newcastle-Ottawa Scale.

b SNNPE: Southern Nations, Nationalities, and Peoples of Ethiopia.

### Publication Bias

In this meta-analysis and systematic review, publication bias was assessed using a funnel plot and the Egger test. The funnel plot showed an asymmetrical distribution across the included studies (Multimedia Appendix 3). The Egger test was not statistically significant (*P*=.07), which suggests no publication bias in the included studies. In addition, a nonparametric trim-and-fill analysis was conducted to estimate the overall effect of the imputed studies. The trim-and-fill analysis revealed that no imputed studies affected the overall prevalence of perioperative patient satisfaction (5072/6858, 73.96%, 95% CI 68.84%-79.08%).

### Pooled Prevalence of Perioperative Patient Satisfaction

**Figure 2.**
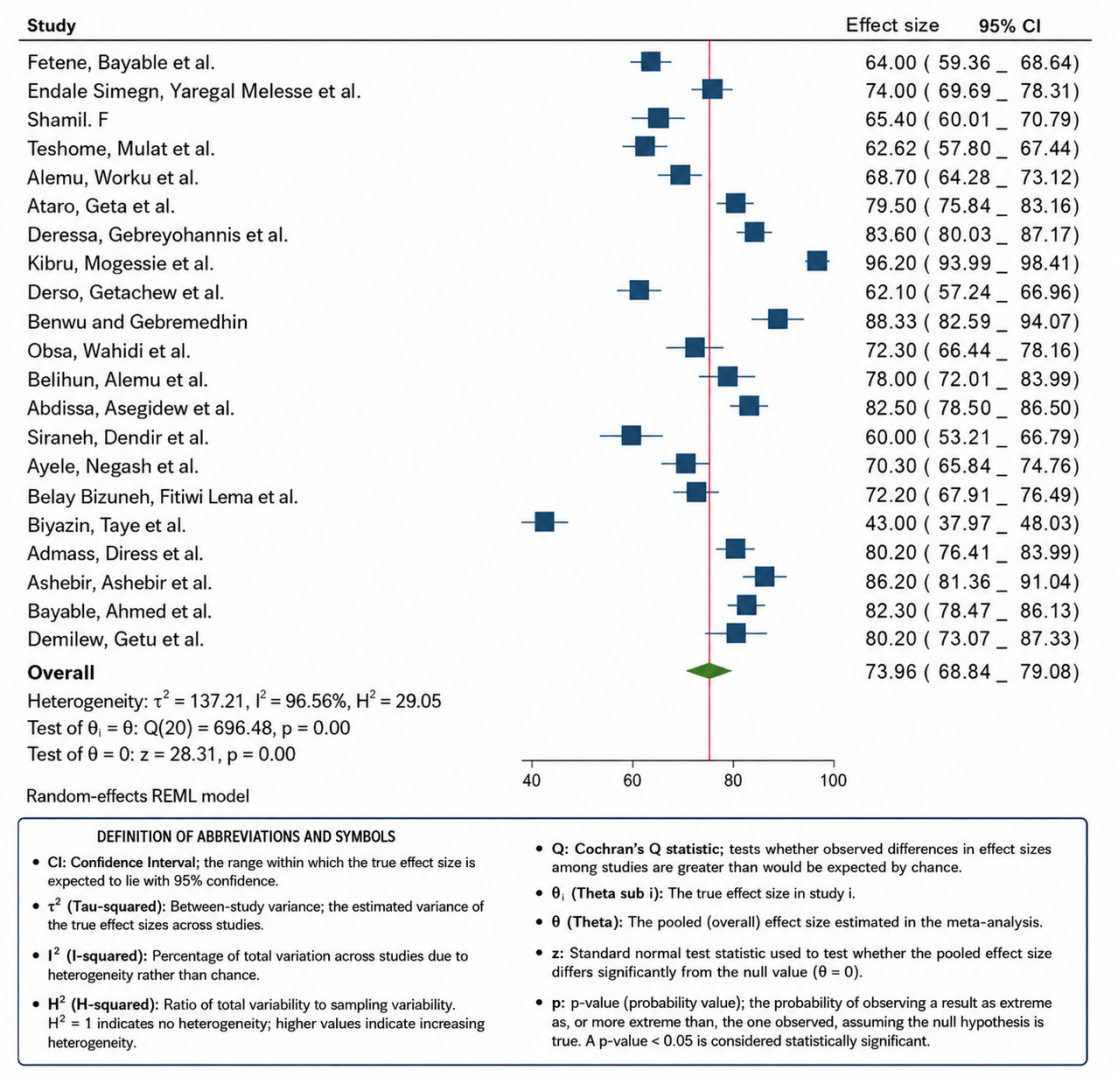
The forest plot was used to assess the pooled prevalence of patient satisfaction with perioperative services [13-33]. REML: restricted maximum likelihood.

In total, 21 studies and 6858 participants were included in this analysis. The pooled prevalence of perioperative patient satisfaction was 73.96% (5072/6858, 95% CI 68.84-79.08) using a random-effects model (*I*^2^=96.56%; *Q*_20_=696.48; *P*<.001) (Figure 2).

### Sensitivity Analysis

A sensitivity analysis was conducted using a random-effects model to identify any outliers or influential studies that affected the overall prevalence of perioperative patient satisfaction. In the analysis, no influential studies were identified, which was confirmed by the fact that all the point estimates remained within the 95% CI (Multimedia Appendix 4).

### Subgroup Analysis

Subgroup analyses revealed significant variation in the prevalence of perioperative patient satisfaction. The overall effect was significant (*P*<.001), with substantial heterogeneity (*I*²=96.56%). Prevalence varied notably by region (Tigray: 106/120, 88.33% vs Oromia: 286/473, 60.46%), publication year (pre-2020: 814/1023, 79.56% vs post-2020: 6099/9519, 73.04%), sample size (<300: 706/899, 78.50% vs ≥300: 6207/9643, 71.75%), sampling technique (nonrandom: 2830/4844, 76.42% vs random: 4083/5698, 71.75%), population (all adult surgical patients: 4191/5591, 75.00% vs elective: 2722/4951, 71.85%), and service type (all perioperative: 3618/4519, 80.07% vs preoperative: 598/967, 61.85%). The overall heterogeneity of this subgroup analysis was *I*²=96.56%, indicating substantial heterogeneity among the subgroup analyses. The meta-regression analysis confirmed that there was no statistically significant association between the pooled effect size and any of the predefined study-level covariates, including publication year, study area, sample size, type of service provided, study population, sampling technique, or assessment tool (Multimedia Appendix 5).

### Meta-Regression Analysis

Meta-regression is used to explore and explain the heterogeneity between study results. It is used to test hypotheses about what factors cause an effect size to be larger in some studies and smaller in others. This systematic review utilized meta-regression to investigate the sources of the considerable heterogeneity (*I*²=96.56%) observed among the included studies. The meta-regression analysis showed no statistically significant associations between the pooled effect size and any of the predefined study-level covariates, including publication year, study area, sample size, types of service provided, study population, sampling technique, or assessment tool (Table 2).

**Table 2.** Univariate meta-regression analysis results for prevalence of patient satisfaction with perioperative services in Ethiopia.

Specific variable	Coefficient (β)	SE	*P* value	95% CI
Year of publication	−2.93	8.62	.73	−19.82 to 13.96
Sampling technique	−7.10	6.25	.26	−19.35 to 5.15
Assessment tool	−4.32	3.16	.17	−10.50 to 1.87
Sample size category	−9.21	5.36	.09	−19.71 to 1.30
Study population	−0.29	3.58	.94	−7.31 to 6.73
Provided service	−3.55	1.93	.07	−7.32 to 0.23
Study area	−3.60	2.82	.20	−9.14 to 1.93

### Factors Affecting Patient Satisfaction With Perioperative Services in Ethiopia

**Figure 3.**
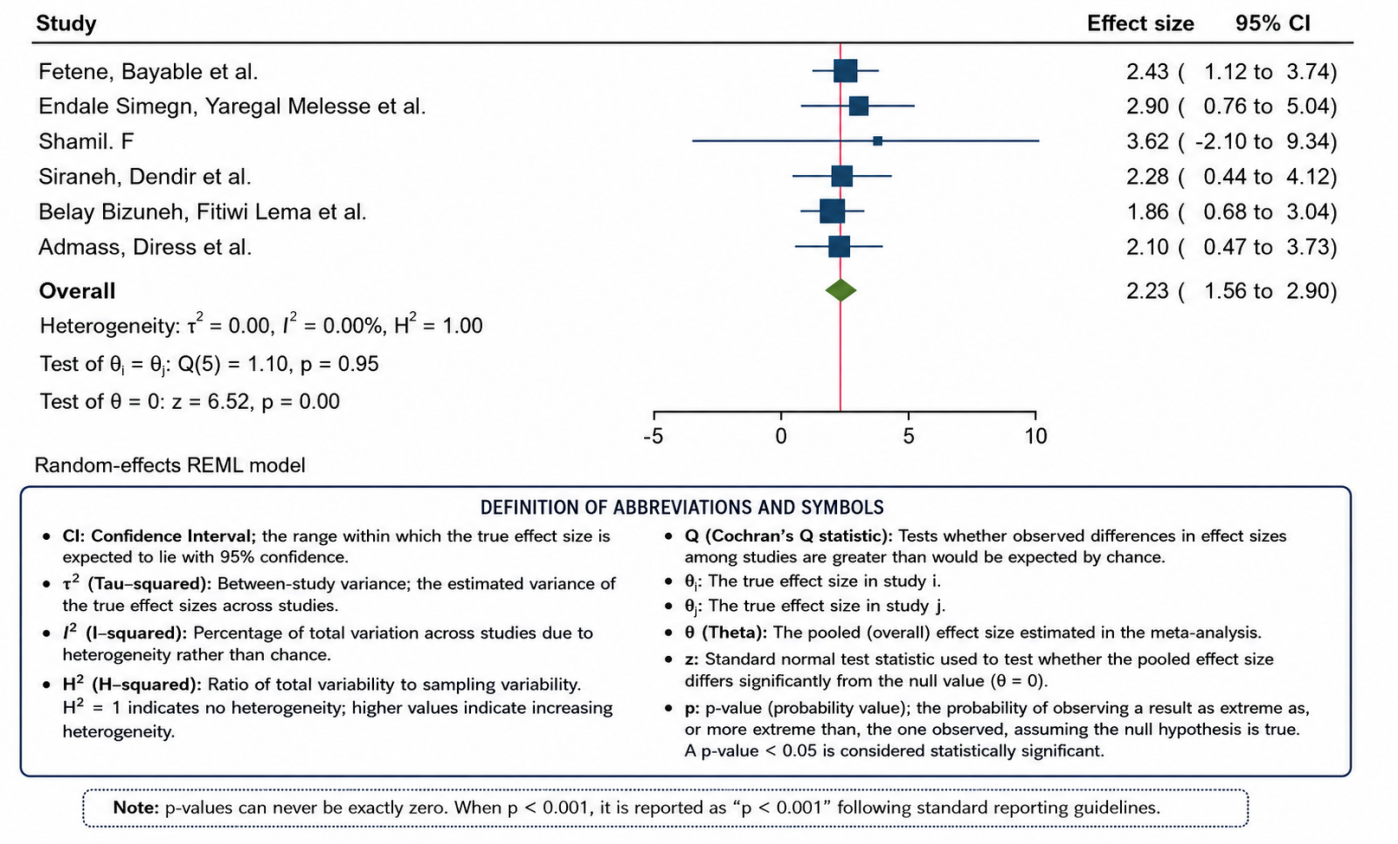
The forest plot was used to assess the association between postoperative pain management and patient satisfaction with perioperative services [13-15,26,28,30]. REML: restricted maximum likelihood.

Patient satisfaction with perioperative services was 2.23 times higher among patients satisfied with effective postoperative pain management than patients who had no postoperative pain management (adjusted odds ratio [AOR] 2.23, 95% CI 1.56-2.90; *I*^2^=0.00%; *Q*_5_=1.10; Figure 3).

**Figure 4.**
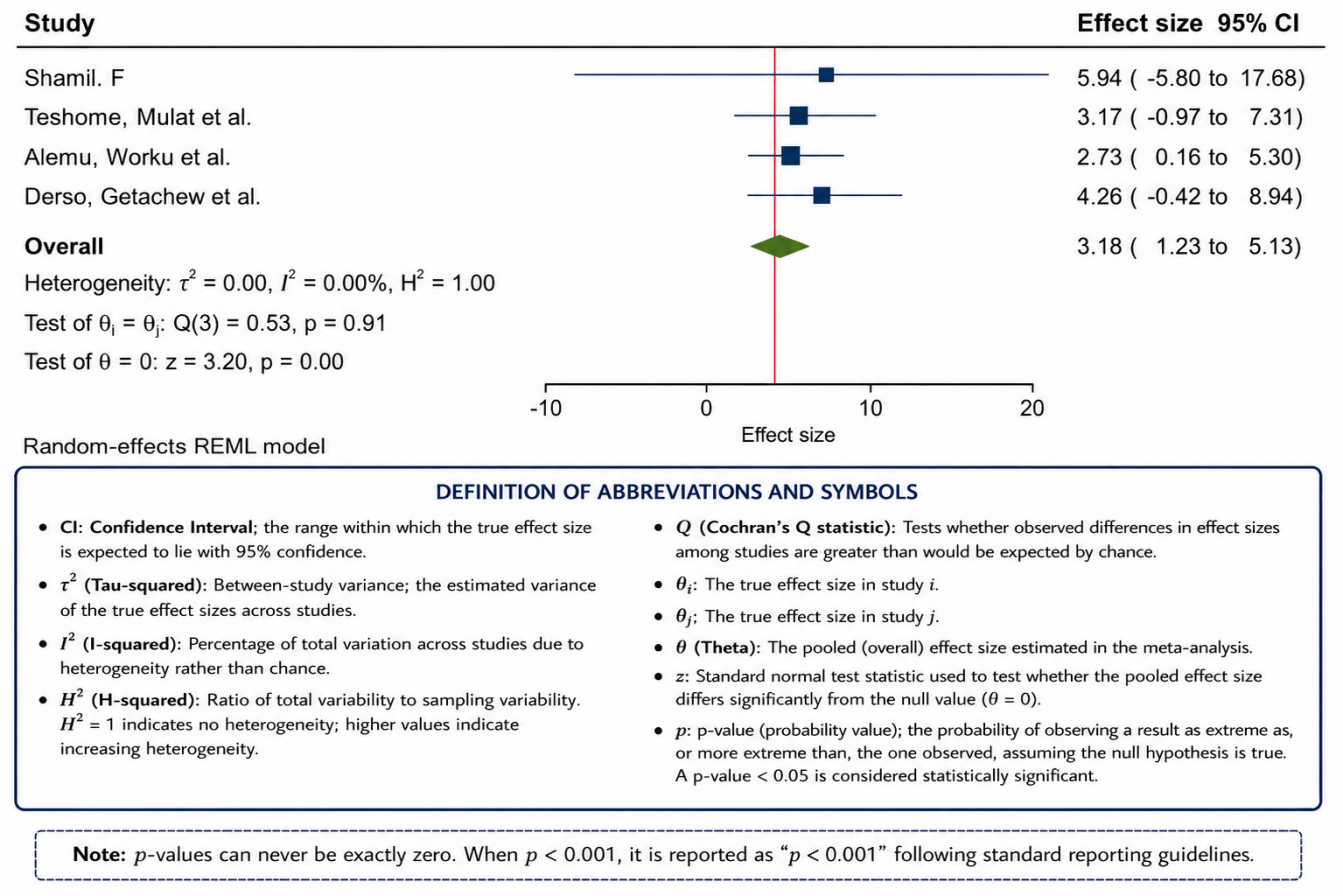
The forest plot was used to assess the association between illiteracy and patient satisfaction with perioperative services [15-17,21]. REML: restricted maximum likelihood.

Satisfaction was 3.18 times greater among illiterate patients than among more educated individuals (AOR 3.18, 95% CI 1.23-5.13; *I*^2^=0.00%; *Q*_3_=0.53; Figure 4).

**Figure 5.**
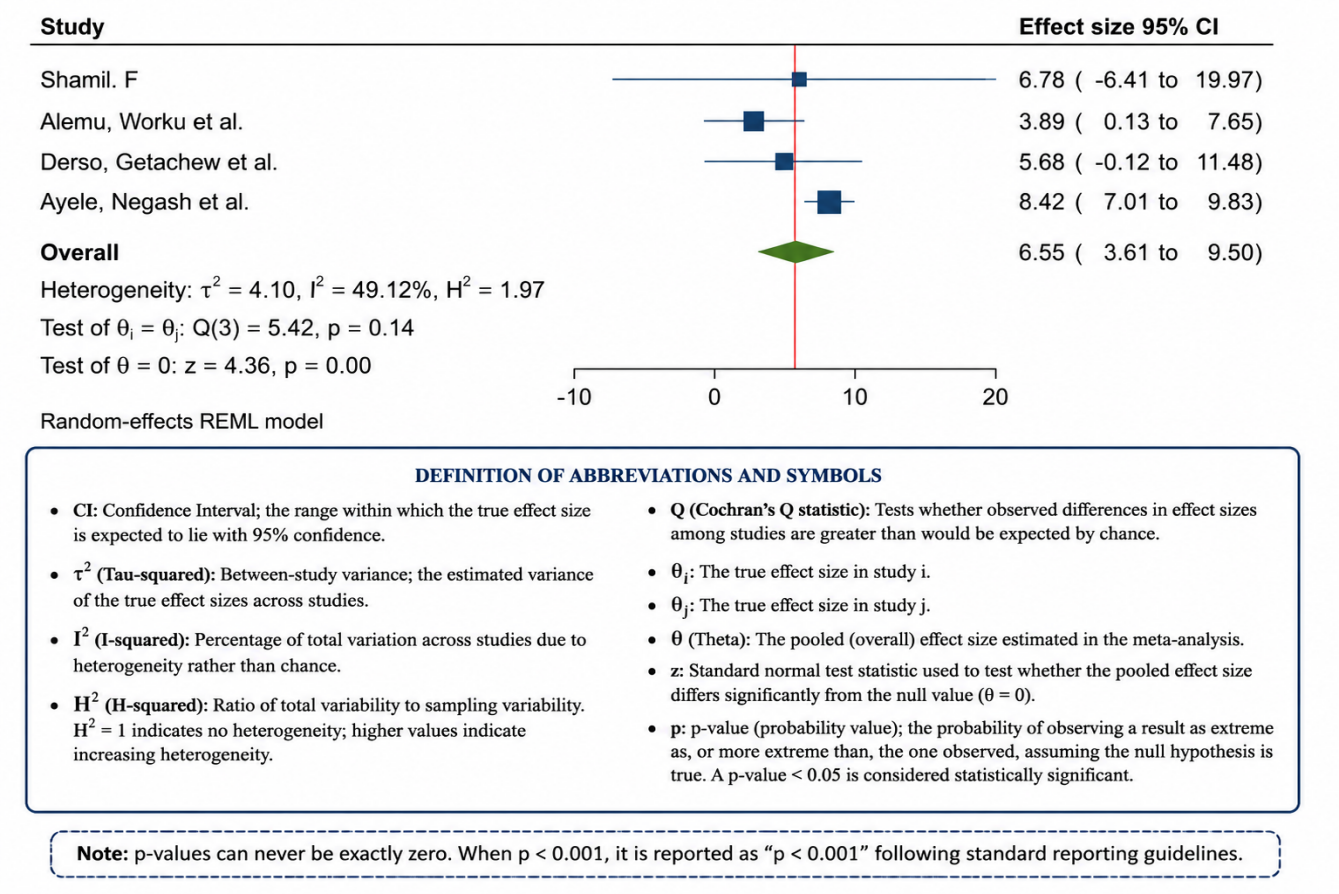
The forest plot was used to assess the association between primary school and patient satisfaction with perioperative services [15,17,21,27]. REML: restricted maximum likelihood.

Patients with a primary school education had 6.55 times higher odds of being satisfied with perioperative services than those with a secondary education or higher (AOR 6.55, 95% CI 3.61-9.49; *I*^2^=49.21%; *Q*_3_=5; Figure 5).

**Figure 6.**
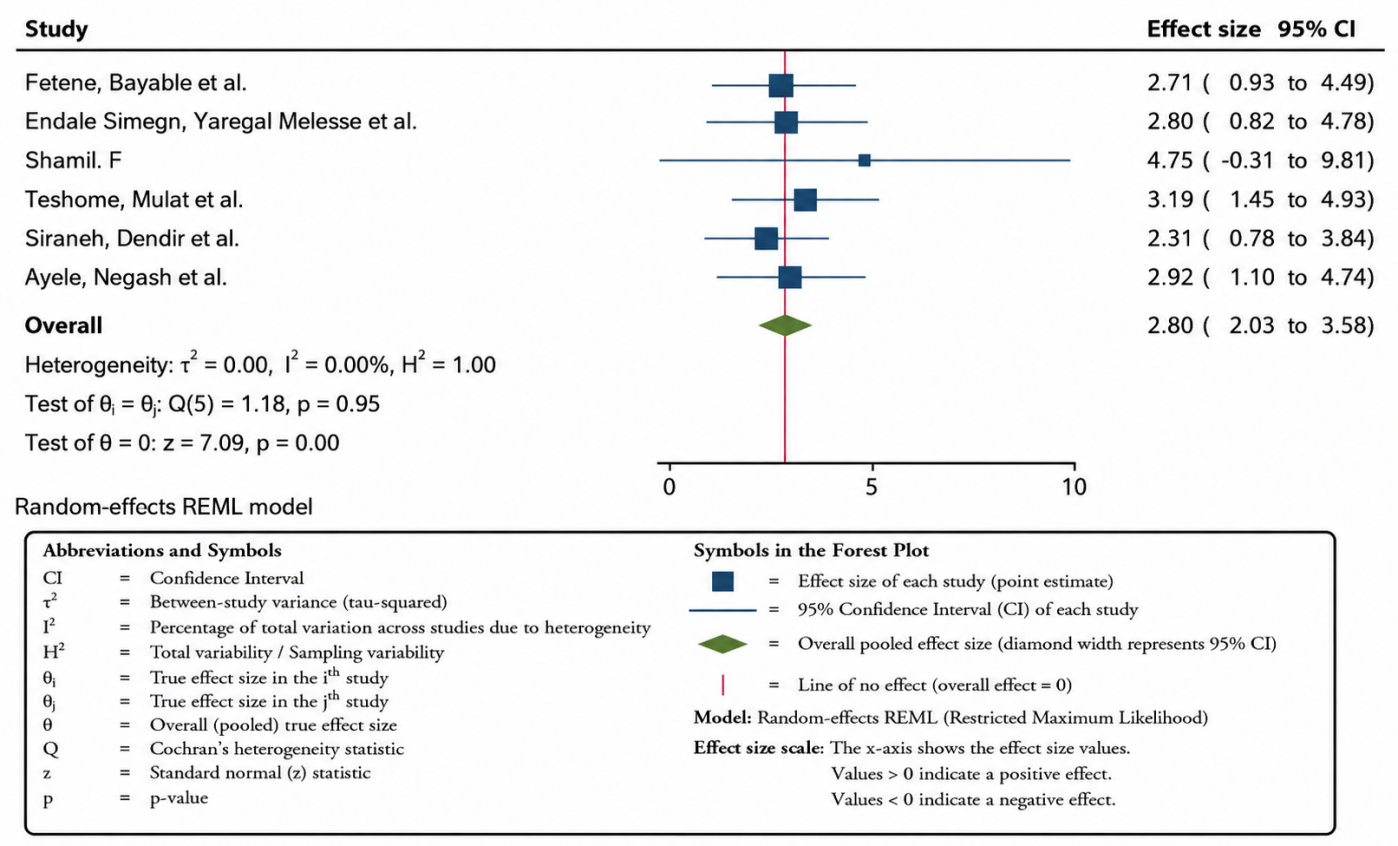
The forest plot was used to assess the association between local anesthesia and patient satisfaction with perioperative services [13-16,26,27]. REML: restricted maximum likelihood.

Patients who received local anesthesia were 2.80 times more likely to be satisfied with perioperative services than those who received general anesthesia (AOR 2.80, 95% CI 2.03-3.57; *I*^2^=0.00%; *Q*_5_=1.19; Figure 6).

**Figure 7.**
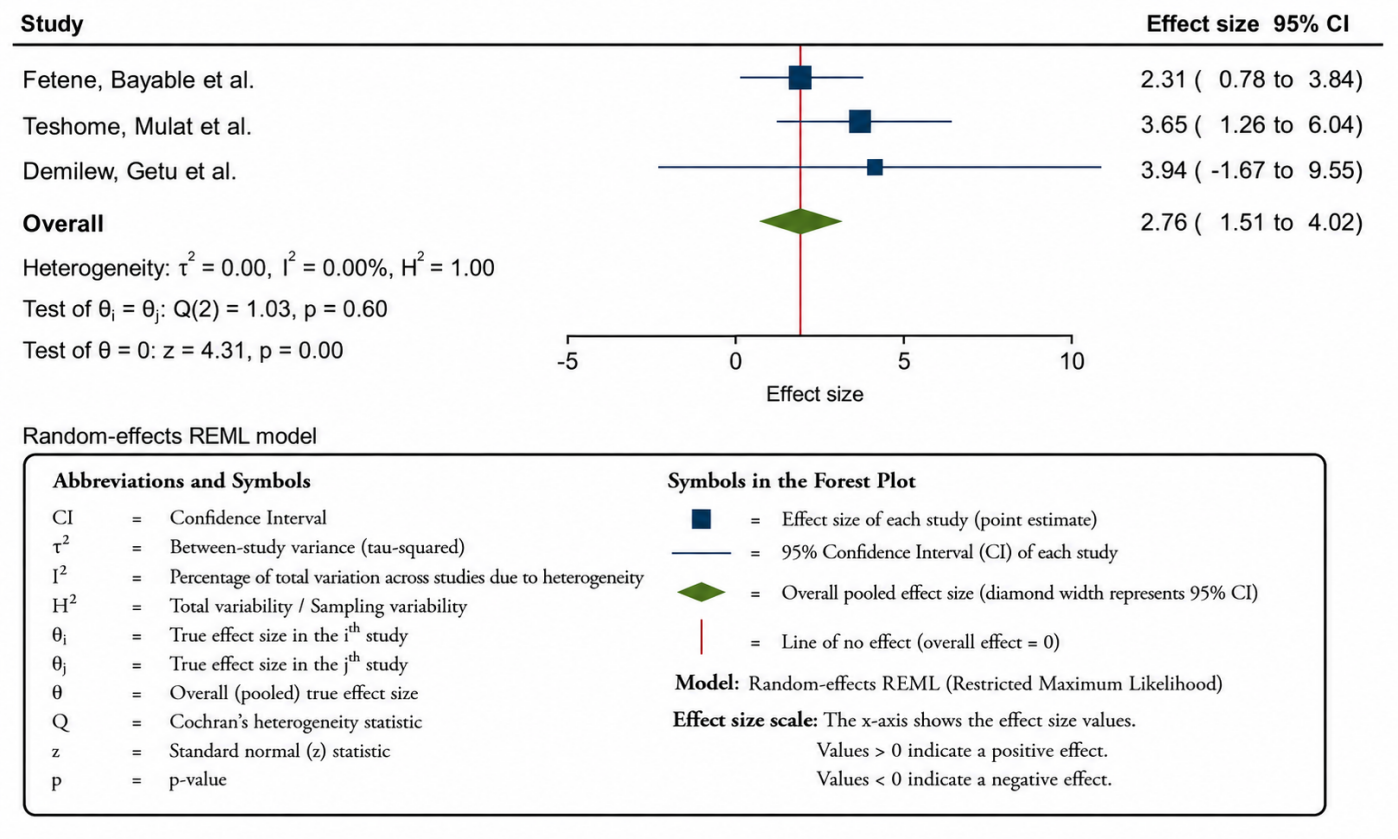
The forest plot was used to assess the association between a previous history of anesthesia or surgery and patient satisfaction with perioperative services [13,16,33]. REML: restricted maximum likelihood.

Patients with a history of surgery or anesthesia were 2.76 times more likely to be satisfied with perioperative services than those without previous surgery or anesthesia (AOR 2.76, 95% CI 1.51-4.01; *I*^2^=0.00%; *Q*_2_=1.03; Figure 7).

## Discussion

### Principal Findings

This systematic review and meta-analysis synthesized data from 21 studies comprising 6858 participants to estimate the pooled prevalence of patient satisfaction with perioperative services in Ethiopia and identify key determinants of satisfaction. This study is significant because it provides valuable insights into patient satisfaction and informs future approaches to enhance patient well-being and recovery.

The analysis revealed that the pooled prevalence of patient satisfaction was 73.96% (5072/6858, 95% CI 68.84%-79.08%), indicating that more than one-quarter of patients were not fully satisfied with their care. This finding was associated with substantial heterogeneity (*I*²=96.56%; *Q*_20_=696.48; *P*<.001). This extremely high heterogeneity indicates that the estimated prevalence should not be interpreted as a single, precise national figure but rather as an overall exploratory estimate. The meta-regression analysis was used to assess the effects of the measured covariates: publication year, study area, and sample size. It suggested that unmeasured factors such as the quality of patient care, patient demographics, or hospital type might be the primary drivers of the variation between studies. Despite this heterogeneity, the pooled estimate is remarkably consistent with findings from other nations, including Nigeria (71%) [39], Portugal (74.5%) [40], and Saudi Arabia (73.5%) [41]. Possible reasons for this consistency could be comparable quality of perioperative services, similar health care challenges and systems, and ongoing health care improvement initiatives in these countries.

Patient satisfaction was greater in the included studies than in studies conducted at Sohag University Hospital (61.9%) [12], in Rwanda (67.43%) [42], at Mount Sinai Hospital (60%) [43], and in the United Kingdom (86.7%) [44]. This discrepancy might be related to disparities in health care systems, including resource access and quality of care; differences in patients’ demographics and health status; methodological variations including differences in study design, patient recruitment methods, and assessment tools; cultural perceptions of health care and patient-provider relationships; and focus on patient-centered care. Patient satisfaction was lower in the included studies than in studies conducted in tertiary hospitals in Cameroon (39/45, 86.7%) [45], Nigeria (94/108, 86.85%) [46], the United Kingdom (4595/4709, 97%) [47], and Australia (11067/11,410, 96.8%) [7]. This discrepancy could be due to variations in the quality and availability of infrastructure, resource allocation (staffing levels, medical equipment, and facilities), cultural expectations and experiences, methodological differences, and health care quality initiatives.

The subgroup analysis revealed several factors contributing to the heterogeneity observed across studies. The higher prevalence of patient satisfaction observed in the Tigray Region may be attributed to the limited number of studies conducted there, which potentially introduced biases into the pooled estimate. Studies published before 2020 reported higher satisfaction levels compared to those published afterward. This shift may be explained by greater access to health-related information in recent years, which has raised patient expectations. When these heightened expectations are not met, satisfaction may decline. Studies with sample sizes below 300 participants showed higher satisfaction than those with 300 or more participants. Smaller samples are often more prone to selection and publication biases and tend to involve more homogeneous populations, which may yield inflated satisfaction estimates. In contrast, larger samples capture greater clinical diversity, often resulting in lower reported satisfaction.

Regarding sampling methodology, nonrandom sampling was associated with higher satisfaction than random sampling. This discrepancy likely reflects selection bias, as nonrandom methods allow researchers to preferentially include individuals with positive experiences, thereby inflating satisfaction rates. Satisfaction was higher among all adult surgical patients compared to those undergoing elective surgery alone. This difference may be explained by variations in surgical urgency, patient expectations, and the overall perioperative experience. Differences in study population also contributed to variation in satisfaction estimates. Patient satisfaction was greater when all perioperative services were considered together than when preoperative services were examined alone. This may be due to the comprehensive nature of full perioperative care, including timely support, effective postoperative pain management, and better alignment with patient expectations.

The findings showed that perioperative patient satisfaction was 2.23 times greater among patients with effective postoperative pain management than those without. This finding was supported by studies conducted in Portugal [40], the United States [48], and Eritrea [11]. This could be due to effective postoperative pain management leading to smoother recovery. Patients frequently associate effective pain relief with high-quality care, which results in more positive experiences and significantly impacts patients’ emotional and psychological well-being.

Patient satisfaction with perioperative services was 3.18 times greater among illiterate patients than among those with higher education. Patient perioperative satisfaction was 6.55 times higher among those in primary school than among those in secondary school and above. A study conducted in Saudi Arabia supported this finding [49]. It is likely that less education is not itself a cause of satisfaction but that it is a proxy for other factors (eg, lower expectations, different communication styles, or fear of authority leading to reported satisfaction). Illiterate patients may have lower expectations about their surgical experience, are less likely to evaluate their care critically, and may benefit more from emotional support and reassurance during the process. This finding frames illiteracy not as a positive factor but as a potential indicator of health literacy gaps and a need for better patient communication and empowerment to ensure that satisfaction reports reflect true care quality rather than perceived power dynamics.

Patients who received local anesthesia were 2.80 times more satisfied with perioperative services than those who received general anesthesia. This is congruent with the results of a study carried out in Eritrea [11]. A possible explanation is that local anesthesia tends to reduce anxiety in patients, as they remain conscious during the procedure. It also allows for quicker recovery, enabling them to return to normal activities sooner. In addition, it has fewer side effects, such as grogginess or nausea, and involves less invasive methods, all contributing to the safety and effectiveness of the procedure. Patients with a history of surgery or anesthesia were 2.76 times more satisfied with perioperative services than those without such a history. This result was consistent with a study carried out in Saudi Arabia [49]. The possible reason is that patients with a history of surgery or anesthesia may have a clearer understanding of the perioperative process, greater trust in health care providers, more active participation in care discussions, familiarity with procedures, and realistic expectations, as their past experiences shape their perceptions of quality and care.

### Strengths and Limitations

In this study, we performed a systematic review and meta-analysis, along with subgroup, meta-regression, and sensitivity analyses. However, this work included only cross-sectional studies, which may limit the ability to establish causal relationships, and the included studies exhibited a high level of heterogeneity. This study also did not include a trial sequential analysis. These limitations should be considered when interpreting the findings.

### Conclusions and Recommendations

Overall patient satisfaction with perioperative services in Ethiopia was good. Significant factors influencing perioperative patient satisfaction include effective postoperative pain management, patients’ educational background, use of local anesthesia, and patients’ previous experiences with surgery or anesthesia. To further improve patient satisfaction, health care facilities should prioritize comprehensive pain management strategies, ensure the delivery of clear and accessible information about perioperative services, and invest in ongoing training for surgical and anesthesia teams. These steps are essential for enhancing perioperative patient satisfaction and quality of patient care in Ethiopia.

### Study Implications for Policymakers

Focusing on comprehensive postoperative pain management strategies, increasing patient awareness and access to information about perioperative services, and investing in ongoing training for surgical and anesthesia teams are crucial steps. Policymakers can significantly advance the quality of perioperative services and enhance overall patient satisfaction with the health care system by adopting these measures.

## Supplementary material

10.2196/84457 Multimedia Appendix 1 Full electronic search strategy.

10.2196/84457 Multimedia Appendix 2 Methodological quality assessment of cross-sectional studies using a modified Newcastle-Ottawa Scale.

10.2196/84457 Multimedia Appendix 3 Funnel plot for the assessment of publication bias for the pooled prevalence of patient satisfaction with perioperative service.

10.2196/84457 Multimedia Appendix 4 The result of the sensitivity analysis conducted on patient satisfaction toward perioperative services and associated factors in Ethiopia.

10.2196/84457 Multimedia Appendix 5 Subgroup analysis to assess heterogeneity in the study of patient satisfaction toward perioperative service and associated factors in Ethiopia. K is the number of studies.

10.2196/84457 Checklist 1 PRISMA 2020 checklist.

## Abbreviations

AOR: adjusted odds ratio
PRISMA: Preferred Reporting Items for Systematic Reviews and Meta-Analyses
