# Supplementary material for: Patient Satisfaction With Perioperative Services and Associated Factors in Ethiopia: Systematic Review and Meta-Analysis
Source: JMIR Perioper Med. 2026 Aug 7;9:e84457. doi: 10.2196/84457 (PMC13450891; doi:10.2196/84457)
Supplement: Supplementary file 2 [file periop-v9-e84457-s001.docx]

Full Electronic Search Strategy for Systematic Review on Patient Satisfaction with Perioperative Services and Associated Factors in Ethiopia

The complete search strategies for all databases are listed below. Searches were executed on January 1, 2024. All searches were limited to human studies and the publication years up to January 1, 2024. The strategy was first developed for PubMed/MEDLINE and then adapted for other databases using appropriate syntax and controlled vocabulary.

**1**. PubMed/MEDLINE

Records Retrieved: 186

Search terms used; ("Patient Satisfaction"[Mesh] OR "patient satisfaction"[tiab] OR satisfied[tiab] OR experience*[tiab]) AND ("Perioperative Care"[Mesh] OR "Preoperative Care"[Mesh] OR "Intraoperative Care"[Mesh] OR "Postoperative Care"[Mesh] OR "Surgical Procedures, Operative"[Mesh] OR perioperative[tiab] OR pre-operative[tiab] OR preop[tiab] OR intraoperative[tiab] OR intra-op[tiab] OR postoperative[tiab] OR post-op[tiab] OR surg*[tiab] OR operat*[tiab] OR anesthe*[tiab]) AND ("Ethiopia"[Mesh] OR Ethiopia*[tiab]”).

2. HINARI

Records Retrieved: 62

Search term used “Patient satisfaction with perioperative service and associated factors, full Text Online, scholarly & peer-reviewed journal, article in Ethiopia”.

3. Cochrane Library

Records Retrieved: 10

Search strategy: ("patient satisfaction"[tiab,kw] OR satisfied[tiab,kw] OR experience*[tiab,kw] OR [mh "Patient Satisfaction"]) AND (perioperative[tiab,kw] OR pre-operative[tiab,kw] OR preoperative[tiab,kw] OR intraoperative[tiab,kw] OR postoperative[tiab,kw] OR surg*[tiab,kw] OR operat*[tiab,kw] OR anesthe*[tiab,kw] OR "operating room"[tiab,kw] OR [mh "Perioperative Care"] OR [mh "Preoperative Care"] OR [mh "Intraoperative Care"] OR [mh "Postoperative Care"] OR [mh "Surgical Procedures, Operative"]) AND (Ethiopia*[tiab,kw] OR [mh Ethiopia]).

4. Web of Science Core Collection

Records Retrieved: 5

Search strategy: TS=("patient satisfaction" OR satisfied OR experience*) AND TS=(perioperative OR pre-operative OR preoperative OR intraoperative OR postoperative OR surg* OR operat* OR anesthe*) AND TS=(Ethiopia*)

5. Scopus

Records Retrieved: 7

Search strategy: ( TITLE-ABS-KEY ( "patient satisfaction" OR satisfied OR experience* ) ) AND ( TITLE-ABS-KEY ( perioperative OR pre-operative OR preoperative OR intraoperative OR postoperative OR surg* OR operat* OR anesthe* OR "operating room" ) ) AND ( TITLE-ABS-KEY ( Ethiopia* ) )

6. African Journals Online (AJOL)

Records Retrieved: 3

Search Strategy Notes: AJOL has limited advanced search capabilities. Multiple simple searches were conducted and results combined: patient satisfaction" AND perioperative AND Ethiopia”, "patient satisfaction" AND surgery AND Ethiopia, and “satisfaction AND preoperative AND Ethiopia”

7. Additional Grey Literature & Sources from Ethiopian research repositories

Institutional Repository: Searched for theses and dissertations from Addis Ababa University, Jimma University, Bahr Dar University, Hawasa University and Gonder University research repositories.

Records Retrieved: 5

Note; the reference lists of all included studies and relevant review articles were hand-searched for additional eligible studies. Forward citation tracking was performed for key included studies using Google Scholar.

Total Records Identified from All Sources (Before Deduplication): ~216 (Sum of retrieved records from databases and targeted grey literature screening).
