# Supplementary material for: Patient Satisfaction With Perioperative Services and Associated Factors in Ethiopia: Systematic Review and Meta-Analysis
Source: JMIR Perioper Med. 2026 Aug 7;9:e84457. doi: 10.2196/84457 (PMC13450891; doi:10.2196/84457)
Supplement: Supplementary file 3 [file periop-v9-e84457-s002.docx]

| **First author, publication year** | Criteria | | | | | | | |  |
| --- | --- | --- | --- | --- | --- | --- | --- | --- | --- |
|  | **Selection** | | | | **Comparability** | | **Outcome** | |  |
|  | Representativeness of the sample | Sample size | Non –respondents | Ascertainment of exposure/risk factor | Study controls most important factor | Study controls any additional factor | Assessment of the outcome | Statistical test | **Total score**  **(10)** |
| **Fetene, Bayable et al. 2022** | **B*** | **A*** | **A*** | **B*** | **A*** | **B*** | C* | **A*** | **8** |
| **Endale Simegn, Yaregal Melesse et al. 2021** | **A*** | **A*** | **A*** | **B*** | **A*** | **B*** | C* | **A*** | **8** |
| **Shamil 2021** | **B*** | **A*** | **A*** | **B*** | **A*** | **B*** | B** | **A*** | **9** |
| **(Teshome, Mulat et al. 2022** | **A*** | **A*** | **A*** | **A**** | **A*** | **B*** | C* | **A*** | **9** |
| **Alemu, Worku et al. 2023** | **B*** | **A*** | **A*** | **B*** | **A*** | **B*** | C* | **A*** | **8** |
| **Ataro, Geta et al. 2024** | A* | **A*** | **A*** | **B*** | **A*** | **B*** | A** | **A*** | **9** |
| **(Deressa, Gebreyohannis et al. 2022** | A* | **A*** | **A*** | **A**** | **A*** | **B*** | C* | **A*** | **9** |
| **Kibru, Mogessie et al. 2023** | B* | A* | A* | B* | A* | B* | C* | A* | **8** |
| **Derso, Getachew et al. 2024** | A* | A* | A* | B* | A* |  | C* | A* | **7** |
| **Benwu and Gebremedhin 2019** | A* | A* | A* | B* | A* | B* | A** | A* | **9** |
| **Belihun, Alemu et al. 2015** | A* | A* | A* | B* | A* | B* | A** | A* | **9** |
| **Obsa, Wahidi et al. 2016** | A* | A* | A* | B* | A* | B* | C* | A* | **8** |
| **Abdissa, Asegidew et al. 2023** | A* | A* | A* | B* | A* | B* | B** | A* | **9** |
| **Siraneh, Dendir et al. 2020** | B* | A* | A* | A** | A* | B* | A** | A* | **10** |
| **Ayele, Negash et al. 2022** | B* | A* | A* | B* | A* |  | A** |  | **7** |
| **Belay Bizuneh, Fitiwi Lema et al. 2020** | A* | A* | B | B* | A* | B* | C* | A* | **7** |
| **Biyazin, Taye et al. 2022** | C | A* | A* | A** | A* | B* | A** | A* | **9** |
| **Admass, Diress et al. 2024** | A* | A* | A* | B* | A* | B* | C* | A* | **8** |
| **Ashebir, Ashebir et al. 2024** | A* | A* | A* | B* | A* | B* | B** | A* | **9** |
| **Bayable, Ahmed et al. 2020** | B* | A* | A* | B* | A* | B* | C* | A* | **8** |
| **Demilew, Getu et al. 2021** | A* | A* | A* | B* | A* |  | C* | A* | **7** |

*Note: from each item account point. (Accept the study if total score ≥5)*

NEWCASTLE - OTTAWA QUALITY ASSESSMENT SCALE (adapted for cross sectional studies)

**Selection: (Maximum 5 stars)**

1) Representativeness of the sample:

a) Truly representative of the average in the target population. * (all subjects or random sampling)

b) Somewhat representative of the average in the target population. * (nonrandom sampling)

c) Selected group of users.

d) No description of the sampling strategy.

2) Sample size:

a) Justified and satisfactory. *

b) Not justified.

3) Non-respondents:

a) Comparability between respondents and non-respondents characteristics is established, and the response rate is satisfactory. *

b) The response rate is unsatisfactory, or the comparability between respondents and non-respondents is unsatisfactory.

c) No description of the response rate or the characteristics of the responders and the non-responders.

4) Ascertainment of the exposure (risk factor):

a) Validated measurement tool. **

b) Non-validated measurement tool, but the tool is available or described.*

c) No description of the measurement tool.

**Comparability: (Maximum 2 stars)**

1) The subjects in different outcome groups are comparable, based on the study design or analysis. Confounding factors are controlled.

a) The study controls for the most important factor (select one). *

b) The study control for any additional factor. *

**Outcome: (Maximum 3 stars)**

1) Assessment of the outcome:

a) Independent blind assessment. **

b) Record linkage. **

c) Self report. *

d) No description.

2) Statistical test:

a) The statistical test used to analyze the data is clearly described and appropriate, and the measurement of the association is presented, including confidence intervals and the probability level (p value). *

b) The statistical test is not appropriate, not described or incomplete.

This scale has been adapted from the Newcastle-Ottawa Quality Assessment Scale for cohort studies to perform a quality assessment of cross-sectional studies for the systematic review.

References

Peterson J, Welch V, Losos M, Tugwell P. The Newcastle-Ottawa Scale (NOS) for Assessing the Quality of Nonrandomised Studies in Meta-Analyses. Vol 2. Ottawa Hospital Research Institute; 2011:1-12.
